# 3D Superparamagnetic Scaffolds for Bone Mineralization under Static Magnetic Field Stimulation

**DOI:** 10.3390/ma12172834

**Published:** 2019-09-03

**Authors:** Irina Alexandra Paun, Bogdan Stefanita Calin, Cosmin Catalin Mustaciosu, Mona Mihailescu, Antoniu Moldovan, Ovidiu Crisan, Aurel Leca, Catalin Romeo Luculescu

**Affiliations:** 1 Center for Advanced Laser Technologies (CETAL), National Institute for Laser, Plasma and Radiation Physics, RO-077125 Magurele-Ilfov, Romania; 2 Physics Department, Faculty of Applied Sciences, University Politehnica of Bucharest, RO-060042 Bucharest, Romania; 3 Horia Hulubei National Institute for Physics and Nuclear Engineering IFIN-HH, RO-077125 Magurele-Ilfov, Romania; 4 National Institute for Laser, Plasma and Radiation Physics, RO-077125 Magurele-Ilfov, Romania; 5 National Institute of Materials Physics, RO-077125 Magurele-Ilfov, Romania

**Keywords:** superparamagnetic scaffold, composite, laser direct writing, static magnetic field, extracellular matrix mineralization

## Abstract

We reported on three-dimensional (3D) superparamagnetic scaffolds that enhanced the mineralization of magnetic nanoparticle-free osteoblast cells. The scaffolds were fabricated with submicronic resolution by laser direct writing via two photons polymerization of Ormocore/magnetic nanoparticles (MNPs) composites and possessed complex and reproducible architectures. MNPs with a diameter of 4.9 ± 1.5 nm and saturation magnetization of 30 emu/g were added to Ormocore, in concentrations of 0, 2 and 4 mg/mL. The homogenous distribution and the concentration of the MNPs from the unpolymerized Ormocore/MNPs composite were preserved after the photopolymerization process. The MNPs in the scaffolds retained their superparamagnetic behavior. The specific magnetizations of the scaffolds with 2 and 4 mg/mL MNPs concentrations were of 14 emu/g and 17 emu/g, respectively. The MNPs reduced the shrinkage of the structures from 80.2 ± 5.3% for scaffolds without MNPs to 20.7 ± 4.7% for scaffolds with 4 mg/mL MNPs. Osteoblast cells seeded on scaffolds exposed to static magnetic field of 1.3 T deformed the regular architecture of the scaffolds and evoked faster mineralization in comparison to unstimulated samples. Scaffolds deformation and extracellular matrix mineralization under static magnetic field (SMF) exposure increased with increasing MNPs concentration. The results are discussed in the frame of gradient magnetic fields of ~3 × 10^−4^ T/m generated by MNPs over the cells bodies.

## 1. Introduction

Bone is the second most commonly transplanted tissue, preceded only by blood transfusion [1]. The cost of osteoporotic fractures is estimated to reach 77 billion euros by 2050 [2]. Within this context, it is imperative to achieve the functional and structural restoration of damaged bone tissue [3,4,5]. A major difficulty in bone tissue engineering arises from the fact that the bone regeneration process requires a long time for achieving a completely functional tissue [6]. Generally, cells are seeded ex vivo into a three-dimensional (3D) biocompatible and sometimes biodegradable structure called scaffold, where they attach and grow. After the implantation into the injured site, the scaffolds should allow proper host cell colonization for regeneration purposes [7,8,9,10].

Magnetic scaffolds emerged as promising solution for this purpose. Activation of the magnetic scaffolds using external static magnetic fields (SMF) prevents the decrease of bone mineral density [11] and promotes the bone regeneration in bone fractures [12]. The significant alterations in cell behaviors stimulated by the externally applied magnetic fields has been demonstrated in numerous studies [8,11]. For example, it has been shown that an externally applied SMF using a magnet accelerates the osteogenic differentiation of osteoblasts-like cells in vitro and triggers peri-implant bone formation in vivo.

The magnetism can also be used through scaffolding materials with magnetic properties. For example, biomaterials that incorporate magnetic nanoparticles (MNPs) are being developed [6,13,14,15,16,17]. The superparamagnetic behavior of the MNPs increased the adhesion and differentiation of osteoblastic cells in vitro and the bone formation in vivo [13,14,15,16,17,18]. Structures with such intrinsic magnetic properties represent a promising biomatrix for bone tissue engineering [13,14,15,16,17,19,20]. It was also shown that the changes in the magnetic properties of MNPs in the presence of a magnetic field had no influence on cellular toxicity [6]. Additionally, magnetic scaffolds with incorporated MNPs increased the mechanical strength of the scaffolds and promoted the osteogenic differentiation of the seeded cells [5,13,14,15]. Moreover, the use of MNPs results in superior physiochemical properties of the material and closer replication of the hierarchical nanostructure of bone tissue [16,17]. Furthermore, the iron metabolism facilitates the proliferation of bone or non-bone cell lines [18,19,20] and has a positive influence on the bone density [21,22].

Previous attempts to fabricate magnetically-active scaffolds employed ceramics, gelatin or polymers that were impregnated with MNPs by freeze drying, deep coating and direct nucleation of fiber deposition [23]. Porous polycaprolactone scaffolds loaded with MNPs stimulated in external SMF promoted the osteoblastic differentiation of primary mouse calvarial osteoblasts [24]. A time-dependent magnetic field applied on 3D cylindrical poly(ε-caprolactone)/iron-doped hydroxyapatite nanocomposite scaffold fabricated by fiber deposition had osteogenic effects on seeded human mesenchymal stem cells [25]. The newest approaches assign the benefic role of the MNPs on the cellular behavior to the existence of high magnetic field gradients that traverse the cell bodies [26,27]. In scaffolds with incorporated MNPs, the nanoparticles concentrate the externally applied magnetic field and produce high gradients magnetic fields across the cells bodies [26,27]. It has been shown that in SMFs with gradients above 10^4^ T/m the magnetic force magnitudes are comparable with the gravitational forces and affect the cell machinery [26,27]. Such magnetic field gradients promote the cell migration to the areas with the strongest magnetic field gradient. In particular, enhanced bone regeneration in osteoblast-like cells seeded on scaffolds with incorporated MNPs has been be explained through the integrins- and bone morphogenetic proteins-mediated signaling pathways, which improve the osteoblasts’ functions and is beneficial for bone formation [24,28]. Despite these advantages, the fabrication of scaffolds containing MNPs for orthopedic applications has been restricted to few studies and the mechanism of action of SMFs on the bone regeneration process remains unknown [29,30]. Furthermore, the composite magnetic scaffolds reported previously provide with no control over the amount of loaded MNPs [31,32].

Currently, the major challenge is to fabricate magnetic scaffolds with reproducible architectures that contain precise MNPs concentrations and have a homogenous distribution of the nanoparticles over the scaffolds’ structure [23,31,32]. In this study, we report a new method for fabricating innovative magnetic scaffolds with incorporated MNPs having unique advantages compared to the scaffolds reported by previous works. Specifically, the scaffolds developed in our study possess fully controllable 3D architectures, the MNPs are distributed in the scaffolds in precise concentrations, they have a homogenous distribution in the whole scaffolds’ structure and preserve their superparamagnetic behavior. The combination of materials (photopolymer and MNPs) and the fact that the photopolymer/MNPs composite is processed by laser direct writing via two photons polymerization represent the original aspects of the work.

The scaffolds were fabricated from photopolymer/MNPs composites by laser direct writing via two photons polymerization (LDW via TPP) and tested in respect with osteogenic potential [33,34,35,36,37]. The photopolymer Ormocore was employed as 3D structurable material because of its biocompatibility and suitability for bone tissue engineering [38,39]. LDW via TPP technique is a sort of 3D printing that creates objects from 3D model data. To date, it has been used for processing magnetic nanocomposites mostly in combination with other techniques, such as electrodeposition and selective electroless magnetite plating [40,41,42]. While it was possible to create structures that demonstrate a proof-of-principle, the results were generally unreliable for practical applications. The scaffolds were seeded with nanoparticle-free osteoblast-like cells and exposed to static magnetic field of 1.3 T. The scaffolds’ ability to control the cells behavior in terms of cells attachment and early extracellular matrix mineralization was assessed. The results were discussed in the frame of high gradient magnetic fields generated by the MNPs over the cells bodies.

## 2. Materials and Methods

### 2.1. Materials

The photopolymer (Ormocore) and the developer (Ormodev) were purchased from Micro resist technology GmbH (Berlin, Germany). The superparamagnetic nanoparticles with 4.9 ± 1.5 nm diameters and maghemite structure (gamma–Fe_2_O_3_) were produced by laser pyrolysis in identical experimental conditions as those reported in [33]. The laser pyrolysis technique relies on the laser-driven heating of an iron precursor in vapor phase in presence of oxygen [33,34]. The experimental parameters used for producing the MNPs used in this study are reported in [33]: laser power (CO_2_ laser) 55 W, beam diameter 1.5 mm, Fe(CO)_5_ flux 19 sccm, carrier gas flux 100 C_2_H_4_ + 70 Air sccm, productivity about 3.3 g/h. The saturation magnetization was 30 emu/g at room temperature, as determined by [34].

### 2.2. Scaffolds Design and Fabrication

Ormocore/MNPs composites were prepared by adding MNPs in Ormocore, in 0, 2 and 4 mg/mL concentrations. The homogeneous dispersion of MNPs in Ormocore viscous liquid formulation is essential for obtaining 3D scaffolds by proposed method. The unpolymerized Ormocore/MNPs composite was homogenized by 1000 W powerful ultrasonicator at 20 kHz (Hielscher Ultrasonics GmbH, Model UIP1000hdT) for about 30 s. Dispersions with MNPs concentrations in Ormocore up to 32 mg/mL showed good stability for several months. The stability of the unpolymerized i.e. liquid Ormocore/MNPs composite is important for the laser direct writing process, since any inhomogeneity of the irradiated material causes irregularities in the morphology of the scaffolds or it can even impede the photopolymerization process. In general, the stability of a dispersion is evaluated on a case-by-case basis, because it depends on how long we need the system to remain stable. In our experimental conditions, the unpolymerized, i.e., liquid Ormocore/MNPs composite only needs to be stable for several minutes, because this is how long the laser direct writing of the scaffolds lasts. Since the evaluation of the long-term stability of a dispersion is a rather complicated process and since we do not need such long time scales for the stability of our dispersions, in our experimental conditions we resumed to monitor the stability of the unpolymerized Ormocore/MNPs composite by visual inspection. For this, drops of unpolymerized composite were placed on glass slides and visualized under the optical microscope of the Nanoscribe system that was able to image any clusters formed by MNPs aggregation.

**Figure 1:**
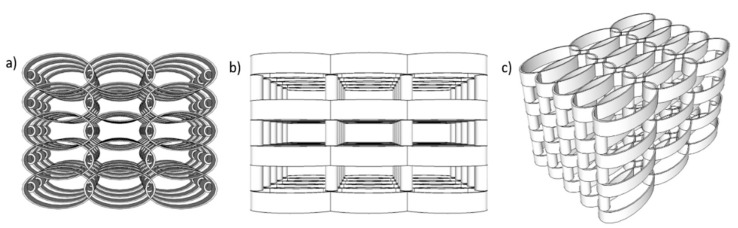
Line plot example of the optimized scaffold design with five layers: (**a**) top view; (**b**) lateral view; (**c**) inclined view.

The scaffolds design was calculated using Python 3.6.6. All information related to structure geometry was delivered as a list of carthesian points, appropriately configured for the 3D lithography installation (Nanoscribe Photonic Professional). The design of the microstructures is presented in Figure 1. As a basis, we started from the optimized geometry reported in [35] that provided suitable porosity and mechanical resilience for the attachment and growth of osteoblast cells. In our recent study [35], we reported that when consecutive layers of ellipsoidal units were not separated on the vertical axis, the cells were not able to penetrate inside the structure of the scaffold and covered only the outer areas. For populating the whole volume of the scaffolds with interconnecting cells, the spacing between neighboring layers had to be increased with respect to the *Z*-axis and this was achieved by separating the consecutive layers of ellipsoidal units using cylindrical pillars.

The scaffolds were fabricated by laser direct writing via two-photon polymerization (LDW via TPP) [36]. The typical processing methodology consists in drop-casting several μL of photopolymerizable material on a glass substrate, followed by laser irradiation and sample development. We used 170 µm thick BK7 glass slides as substrates. The glass slides were cleaned using isopropanol. The Ormocore/MNPs composites were irradiated with 120 fs pulses, with a central wavelength λ = 780 nm, and a frequency of 80 MHz. Both the laser focus and the sample were mobile (sample on X-Y axes, laser beam on Z-axis). For high resolution sample positioning, the laser processing system uses a set of three synchronized piezoelectric stages. After the laser writing, the obtained Ormocore/MNPs composite scaffolds require no additional pre- or post-processing steps other than immersion in Ormodev solution for 3 min, to wash away the non-polymerized material.

### 2.3. Scaffolds Characterization

Scanning Electron Microscopy (SEM): the morphology of the magnetic scaffolds was investigated by Scanning Electron Microscopy (SEM, FEI InspectS model, Thermo Fisher Scientific, Waltham, MA, USA), using a 5 kV voltage. Prior to examination, the scaffolds were coated with a 10 nm layer of gold. Scaffolds shrinkage was calculated as [(bottom area − top area)/bottom area] × 100, where the top and bottom areas were determined from SEM images.

Enhanced Dark-Field Microscopy (EDFM): the location and distribution of the MNPs inside the scaffolds were investigated using CytoViva system (CytoViva Inc., Auburn, AL, USA), without any prior special preparation and in a nondestructive manner. CytoViva comprises a dark-field set illuminator that focuses at diagonal inclinations over the sample and is suitable to investigate translucent materials, based on the scattered light by the nanometric details of the sample. The technique has the capability of high signal-to-noise optical performance based on patent-pending deconvolution and particle location routines providing three dimensional optical image of the sample. The Z stacks images were collected at 100 nm between slices using a 60× oil immersion objective on Q-imaging Exi Blue Charged Coupled Device (CCD) (6.45 × 6.45 μm pixel pitch) at different exposure times, depending on the sample scattering. Two series of stacks (using a piezo-driven Z-axis stage) were acquired for each sample: one with fluorescein (FITC) excited filter with emission at 530 nm, to reconstruct the polymeric structures which are fluorescent at this wavelength, and one in white light, used to locate the nanoparticles in the polymerized Ormocore/MNPs composite.

To process the stacks of images, dedicated plugins were developed by the producer (CytoViva Inc., Auburn, AL, USA), under ImageJ software. The processing procedure started with the synchronization step for all stacks acquired for a given zone of the sample, in order to delimitate the region of interest (which is about 500 × 500 pixels). After this, the processing was different for the stacks acquired in fluorescence (which included the generation of point spread function, iterations for deconvolution until a threshold value was reached, all these being done using parameters like magnification, wavelength, refractive index of immersed oil, x, y, z voxel spacing, mean delta between consecutive iterations) and for the stacks acquired in white light (achieved by using the routine ”Just locate nanoparticles”, establishing the scattered intensity threshold and the number of pixels to represent one nanoparticle). For the investigations, we fabricated the samples in the same conditions as those used for fabricating the scaffolds (MNPs concentration, laser parameters for LDW via TPP process), but with only one layer of ellipses to avoid unnecessary scattering from multiple layers. This did not affect the material behavior or the nanoparticles distribution. The following settings were employed: magnification 60×, pixel dimension 107.5 nm in x–y transversal plane and 100 nm in z direction, oil refractive index 1.516. We maintained the same parameters for all samples. The point spread functions were generated for each stack and the deconvolution routine was run until the mean delta was above 0.001 (for stacks acquired in fluorescence). After that, we generated 3D images only with the ellipsoidal units for each region of interest. The routine “Just locate nanoparticles” was run for all slices, in a stack acquired in white light. It returned 3D images where the MNPs were represented in red and a table with their number and location. Finally, the two 3D images (ellipsoidal units and MNPs respectively) were superposed in ImageJ.

Magnetic Force Microscopy (MFM): the MFM analysis was carried out using a commercial AFM (XE100, Park Systems, Suwon, Korea) with magnetic coated tips (PPP-MFMR, Nanosensors, Thermo Fisher Scientific, Waltham, MA, USA). The MFM images were recorded during a second pass, at a height of 100 nm from the topography scan, using the MFM phase signal. The lift height was selected to be 100 nm because of the specific topography of the samples.

Energy-Dispersive X-ray Spectroscopy (EDS) was performed at 5 kV acceleration voltage inside Scanning Electron Microscopy (FEI InspectS model, Thermo Fisher Scientific, Waltham, MA, USA) using a Si(Li) detector (EDAX Inc., Thermo Fisher Scientific, Waltham, MA, USA). In order to avoid errors in EDS measurements of porous samples/scaffolds, the rectangular structures of 200 × 200 × 20 μm^2^ were fabricated by LDW via TPP of Ormocore/MNPs composites with 0, 2 and 4 mg/mL MNPs concentrations, in identical experimental conditions as the scaffolds. The EDS results are obtained from the average of three different measurements over 40 × 50 μm^2^ areas of polymerized composites, using standardless ZAF analysis. The trace analysis for iron provided errors under 0.5 percent.

Magnetization Measurements have been done using a vibrating sample magnetometer (VSM) module of a Physical Property Measurement System (PPMS) from Quantum Design, Inc., Bucharest, Romania. Initial magnetization versus applied magnetic field as well as major hysteresis loops have been recorded for the scaffolds with MNPs at 300 K in applied magnetic field of up to 5 T. The measurements have been taken with the applied field perpendicular to the scaffold basal plane.

### 2.4. Biological Assessments

Cells seeding: MG-63 osteoblast-like cells were purchased from ECACC (European Collection of Cell Cultures, Salisbury, UK). The cells were cultured in a 25 cm^2^ flask, incubated in an atmosphere of 5% CO_2_ at 37 °C for 24 h and cultured in Minimal Essential Medium, Biochrom containing 10% fetal bovine serum (FBS, Biochrom), 2 mM L-glutamine and 1% non-essential amino acids (complete medium). 100 IU/mL of penicillin/streptomycin was added to the solution. After confluency, the cells were detached with trypsin and seeded on the scaffolds. A cell density of 5000 cells/sample from the 16th cell passage was used. The cells in normal medium were seeded on top of the scaffolds with the aid of a sterile syringe. All chemicals were purchased from Sigma-Aldrich, unnless otherwise specified. Prior cell seeding, the scaffolds were sterilized for 3 h under a UV lamp.

Static Magnetic Field Stimulation (SMF) of the Cell-Seeded Scaffolds: nickel-plated NdFeB rectangular magnets (40 × 40 × 20 mm^3^) with residual magnetism of 1.3 T were purchased from Supermagnete (Gottmadingen, Germany). For SMF stimulation, each cell-seeded scaffold was placed in close vicinity of a magnet. The magnetic stimulation ranged from 3 to 20 days. According to Zablotskii et al., 2016, these timescales of SMF exposure most likely lead to changes at the level of cell shape and size [26]. Control experiments were carried out on scaffolds without SMF exposure. The heating effects in superparamegnetic nanoparticles occur only in the presence of an alternating external magnetic field. Otherwise, like in our experimental conditions where only static magnetic fields are employed, the MNPs act as fillers that reinforce the scaffolds structure and become magnetized only in the presence of the magnetic field, without any thermal effects.

Cells Morphological Investigations: the cell-seeded scaffolds were washed with PBS and fixed for 1 h at 37 °C with 2.5% glutaraldehyde prepared in PBS. The samples were then washed with PBS and dehydrated using a two-steps protocol. In the first step, the samples were dehydrated/washed in ethanol (EtOH) solutions as follows: 2 × 15 min in EtOH 70%, 2 × 15 min in EtOH 90% and 2 × 15 min in EtOH 100%. In the second step, the samples were washed for 3 min in EtOH:HMDS solutions, prepared in 50%:50%; 25%:75% and 0%:100% ratios. Prior to SEM analysis, the samples were left to dry and sputtered with 10 nm of gold. Scanning electron micrographs were recorded with FEI InspectS model. The cells morphology was investigated after 3 days of cultivation. 

Early Mineralization Assay by Alizarin Red S Staining: the cell-seeded scaffolds were analyzed via Alizarin Red S osteogenic differentiation assay that provides qualitative information about the calcium deposits formed in the samples [37]. The cell-seeded scaffolds were washed twice with double-distilled water. Next, 1 mL of 40 mM Alizarin Red S (Sigma Aldrich) (pH 4.1) were added per well. The samples were incubated at room temperature for 20 min and then washed three times with double-distilled water, while shaking. Images of the samples were recorded under a Nikon Eclipse Ti-U microscope equipped with a fluorescence module. The quantification of mineralization was achieved by extracting the calcified mineral at low pH, followed by neutralization with ammonium hydroxide and absorbance measurement at 405 nm. The measurements were performed after 20 days of incubation. 

MTS Assay: 5000 cells/ sample were cultured in complete Minimum Essential Medium (MEM) for 3 days in standard conditions of temperature and humidity. The culture medium was then replaced with 16.67% MTS (Cell Titer 96® Aqueous One Solution Cell Proliferation Assay, Promega) and 83.33% MEM (5% FBS). The supernatant was collected after 3 h of incubation. 100 µL from each sample were distributed in a 96-well plate and the absorbance was measured at 490 nm using a Mitras LB 940 (Berthold Technologies, Bad Wildbad, Germany) spectrophotometer. The viability was calculated as percent from control (cells seeded on glass slides).

Statistical Analysis: for MTS, Alizarin Red Staining (ARS) fluorescence intensity and mineralization assays, the statistical analysis was carried out on five different measurements, with student’s t test, where *p* < 0.05 indicates a significant result.

## 3. Results

### 3.1. Scaffolds Fabrication and Characterization

The scaffolds are composed of elliptical elements of 10 µm in high, disposed in a rectangular matrix. Consecutive levels of ellipses were separated by cylindrical pillars with a diameter of 5 µm and a height of 20 µm. The pillars were placed at the overlap of neighboring elliptical elements on the Y axis (Figure 1).

**Figure 2:**
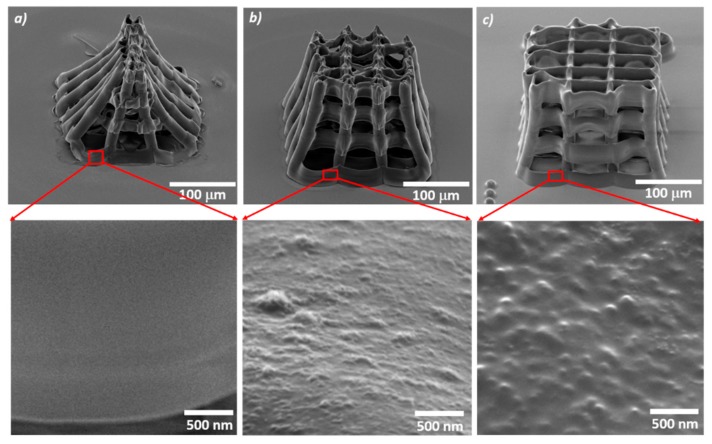
Scanning electron micrographs of scaffolds with magnetic nanoparticles (MNPs) concentrations of (**a**) 0 mg/mL; (**b**) 2 mg/mL; (**c**) 4 mg/mL. Upper panel: scaffolds overview (samples tilted at 45 grd). Lower panel: insets showing close views of the scaffolds’ surfaces.

Figure 2 displays scanning electron micrographs of scaffolds containing different MNPs concentrations. Dispersions with concentrations up to 32 mg/mL and good stability for several months were prepared. However, increasing the concentration of MNPs above 4 mg/mL impeded the photopolymerization process. Most probably, the high density of the MNPs in the photopolymer overheated the material, as proved by extensive bubbles formation followed by local microexplosions in the irradiated volume observed during the laser direct writing process. To provide evidence for this experimental observation, we studied the images recorded with the CCD camera that followed in real time what happened when we irradiated with the focused laser beam the Ormocore/MNPs composite having MNPs concentration above 4 mg/mL (please see the Supplementary information-Figure S1, at the end of the manuscript). There was no trace of polymerized material visible on the glass slide, only the laser spot appears as a small bright zone (Figure S1a). Few seconds later during the laser direct writing process, bubbles were formed (Figure S1b), followed by local micro-explosions of the irradiated material (Figure S1c). Most probably, this happened because the high density of MNPs in the polymer increased significantly the laser absorption at the irradiation spot overheating the material, followed by bubble formation and local micro-explosions. At the end of the laser writing process, no traces of polymerized material were found on the glass slide.

The scaffolds without MNPs underwent a strong shrinkage of top surface and the whole structure reorganized in the shape of a tent (Figure 2a). With increasing MNPs concentrations, the scaffolds’ structural integrity was much improved, indicating that the MNPs play a significant role in structure reinforcement (Figure 2b,c). The shrinkage of scaffolds with different MNPs concentrations are listed in Table 1.

The scaffolds porosities are listed in Table 1 and were determined using Solid Works, similar to as we described in detail in [36]. Except for the scaffolds without MNPs that had porosities below 50% and where the cells were not able to penetrate inside the structure, all the other scaffolds had porosities above 85% that allowed the cell migration from pore to pore. As the structures were highly complex (Figure 1), the pore diameters varied within a broad range, i.e., from 5 to 70 μm.

**Figure 3:**
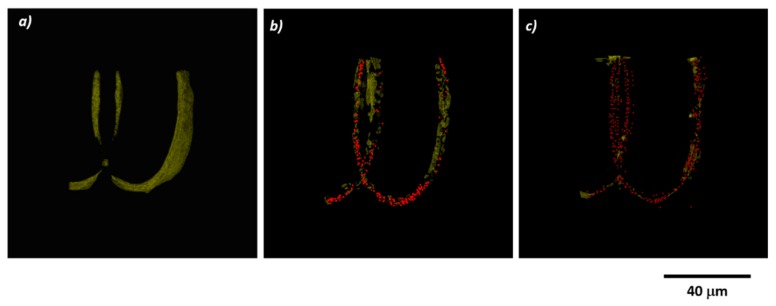
Images obtained by enhanced dark field microscopy using the Cytoviva three-dimensional (3D) module for scaffolds with MNPs concentration of: (**a**) 0 mg/mL; (**b**) 2mg/mL; (**c**) 4 mg/mL.

The location and the spatial distribution of the MNPs inside the scaffolds were monitored by enhanced dark field microscopy (Figure 3). The polymer is represented in yellow and the MNPs as red dots (false colors). To avoid scattering from multiple layers, for this analysis we fabricated particular structures with a single layer of ellipsoidal units. Figure 3b,c show that the MNPs were embedded in the scaffolds and have a homogeneous distribution in the whole volume. The number of nanoparticles from an ellipsoidal unit with 4 mg/mL MNPs concentration was twice the number of nanoparticles from a similar ellipsoid containing 2 mg/mL MNPs (Table 1). This result proves that the polymer/MNPs composites preserved their stoichiometry after the laser direct writing process.

**Figure 4:**
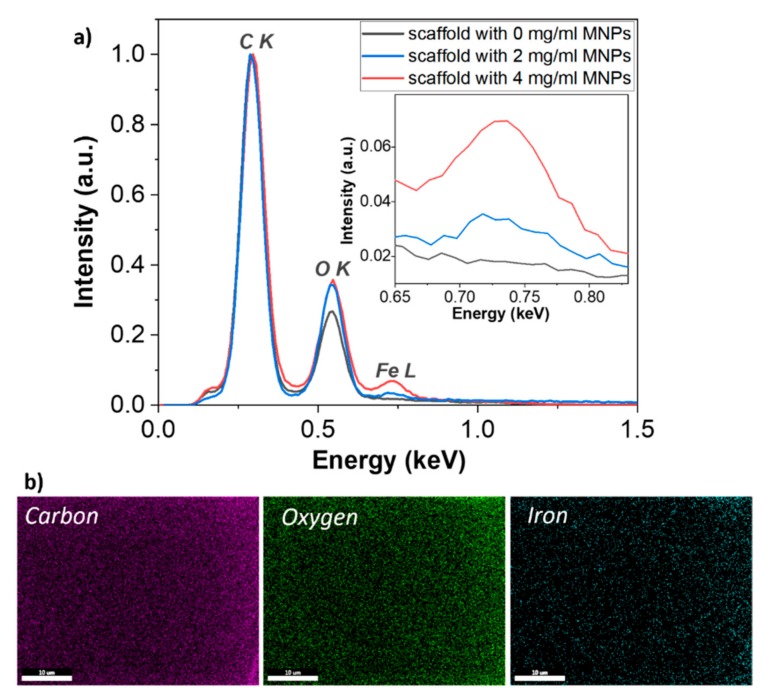
(**a**) Energy-Dispersive X-ray Spectroscopy (EDS) spectra of polymerized Ormocore/MNPs composite with MNPs concentrations of 0, 2 and 4 mg/mL; (**b**) EDS mapping of oxygen, carbon and iron from the polymerized Ormocore/MNPs composite with 4 mg/mL MNPs concentration.

The EDS analysis provided evidence that iron was present in the photopolymerized composites (Figure 4a). The iron was uniformly distributed over the entire investigated areas (Figure 4b), in concordance with the enhanced dark field microscopy findings from Figure 3. Furthermore, the iron concentrations in the polymerized Ormocore/MNPs composites were similar with the MNPs concentrations from the unpolymerized composites (Table 2).

**Figure 5:**
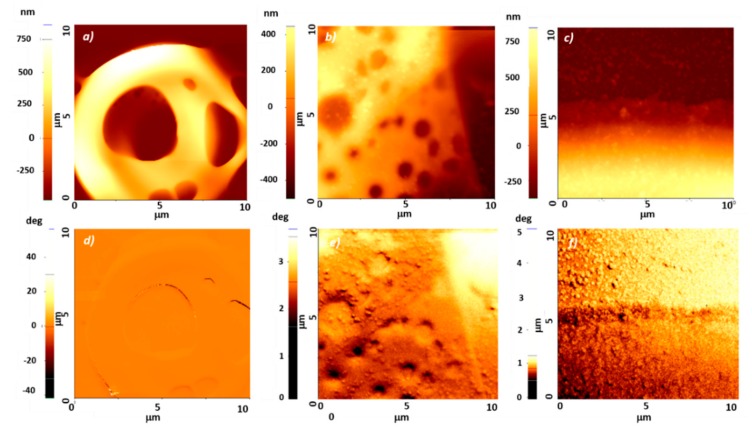
Topographical (upper panel) and magnetic force microscopy (lower panel) images of scaffolds with MNPs concentrations of: (**a**,**d**) 0 mg/mL; (**b**,**e**) 2 mg/mL; (**c**,**f**) 4 mg/mL.

To prove the magnetic nature of the MNPs in the polymerized composites, magnetic force microscopy (MFM) was carried out on scaffolds with different MNPs concentrations (Figure 5). To avoid the magnetic needle to be stacked inside the free spaces of the complex structure of the scaffolds, the analysis was performed on flat areas of the structures. As expected, the topography image of the scaffold without MNPs did not show the presence of any particles (Figure 5a). The surface was locally smooth and continuous and the MFM image revealed only contrast originating from the large topography features. For the scaffolds with 2 and 4 mg/mL MNPs concentrations, the topography images showed the presence of nanoparticles, with a relatively uniform distribution (Figure 5b,c). The particles stand out from the surface between 10 nm and 50 nm. The corresponding MFM images showed some contrast, which can be attributed both to topography effects and to magnetic interaction (Figure 5e,f). A more clear contrast can be noticed in the upper-left part of the MFM image of the magnetic scaffold containing 4 mg/mL MNPs, (Figure 5f), where the particles were spread on the scaffolds surface, with only traces of the embedding polymer.

**Figure 6:**
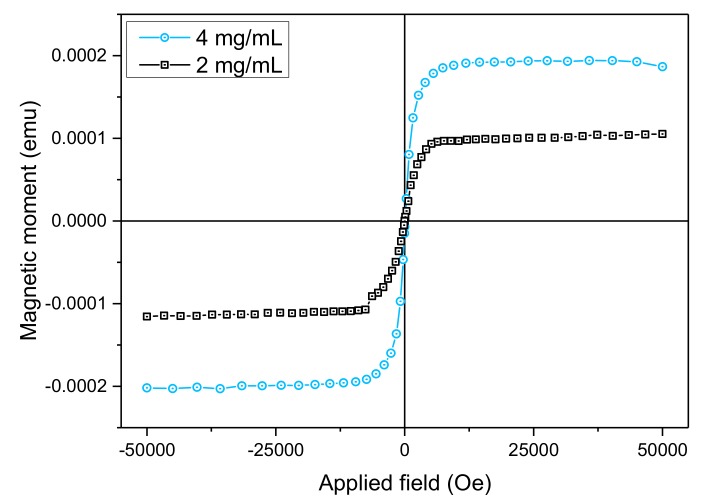
Magnetization versus applied magnetic field (descending branch of the hysteresis loop) for the scaffolds with 2 mg/mL and 4 mg/mL MNPs concentrations, respectively; 1 emu = 10^−3^ Am^2^; 1 Oe = 79.5775 A/m.

The magnetic characteristics of the scaffolds with 2 and 4 mg/mL MNPs concentrations have been investigated using the vibration sample magnetometer (VSM) module of the Physical Property Measurement Systems (PPMS). Full major hysteresis loops were recorded on a single scaffold with MNPs (2 mg/mL and 4 mg/mL, respectively) at 300 K under applied magnetic fields up to 5 T. As there was quite low coercivity (less than 15 Oe) observed for each of the measured scaffolds, we show only the descending branch of the loop. Raw magnetization data have been corrected for the significant diamagnetic signal coming from the scaffold without MNPs. The corrected descending branches of the magnetization versus applied field are shown in Figure 6. A significant magnetic moment of the order of 10^−4^ emu was obtained for both scaffolds. The allure is typical for Fe-rich soft magnetic nanoparticles with fast approach to saturation (both samples saturate at applied fields as low as 9000 Oe), virtually zero remanence and high saturation magnetization. Taking into account the level of doping with MNPs and the single scaffold volume, a total specific magnetization of about 17 emu/g and 14 emu/g for the 4 mg/mL and 2 mg/mL doped scaffolds, respectively, has been determined per single scaffold, in good agreement with the estimated concentrations of nanoparticles per scaffold, listed in Table 1. This estimation is affected by differences between designed and real dimensions of the scaffolds as they are affected by resolution and shrinkage, but nevertheless, it shows that at least half of magnetic moments are preserved during laser photopolymerization.

### 3.2. Biological Assessments

#### 3.2.1. Cells Morphology and Attachment

**Figure 7:**
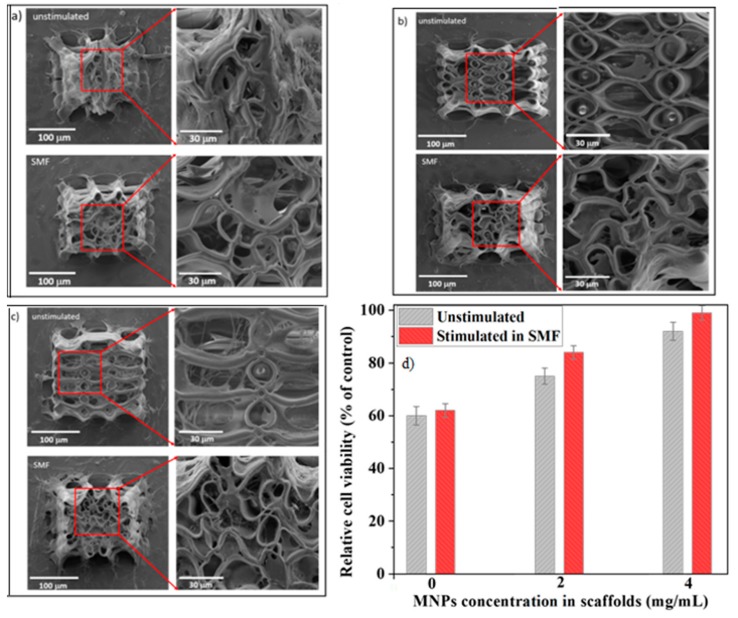
Scanning Electron Microscopy (SEM) micrographs illustrating the cells attached on scaffolds with MNPs concentrations of: (**a**) 0 mg/mL; (**b**) 2 mg/mL; (**c**) 4 mg/mL, after 3 days of cultivation, in the absence (upper panel) and in the presence (lower panel) of static magnetic field (SMF). Left panels: cells growing on the scaffolds. Right panels: insets; (**d**) relative cell viability as a function of MNPs concentration in the scaffolds; except for 0 mg/mL MNPs concentration, the results were statistically significant (*p* < 0.05).

For separating the influence of the scaffolds architecture form that of the magnetic field on the cell behavior, Figure 7a illustrates SEM micrographs of cell-seeded scaffolds without MNPs. Figure 7b,c show SEM micrographs of cells growing on scaffolds with different MNPs concentrations. All SEM images were recorded after 3 days of cultivation, both in the absence and in the presence of SMF. At longer time scales, because of cell growth and division, the morphological insight becomes irrelevant because of the multiple layers of cells overlapping over the entire structure. Several experimental observations are worthy to be mentioned. 

First, on all scaffolds, either exposed or unexposed to SMF, the cells were stretched and had a mature osteoblast phenotype similar with the one from the bone surface. On the scaffolds without MNPs (Figure 7a) the cells mostly grew on the lateral walls of the scaffold, most probably because the tightened scaffolds architecture hampered the cells penetration inside the structure. In contrast, on the scaffolds containing MNPs the cells were able to invade the whole volume of the scaffolds (Figure 7b,c).

Second, the number of cells attached on the scaffolds increased with increasing MNPs concentration. Given that the MNPs are superparamagnetic and therefore activated only in the presence of external SMFs, the increase of the cell attachment with increasing MNPs concentration in scaffolds not exposed to SMF can be ascribed to the nanostructuring of the scaffolds’ surface (insets from Figure 2). This could explain the low number of cells on the scaffolds without MNPs (Figure 7a upper panel), given that these scaffolds have smooth surfaces at nanoscale (inset from Figure 2a). In contrast, the scaffolds with 4 mg/mL MNPs concentration showed numerous cells penetrating inside the scaffolds structure, where they formed an interconnected network (Figure 7c upper panel). Most probably, this happened because their nanostructured surfaces provided more attachment points for the cells (inset from Figure 2c).

A third observation is that, excepting the scaffolds without MNPs, the SMF exposure of the cell-seeded scaffolds caused a dramatic change of the cellular behavior, with more cells attached as compared with the corresponding scaffolds in the unstimulated regime. In addition, the number of attached cells increased with increasing MNPs concentration (Figure 7b,c lower panels). 

Another interesting finding is that, following SMF exposure of the cell-seeded scaffolds containing MNPs, the scaffolds architecture changed dramatically. The initial regular architecture of the scaffolds, comprising of ellipsoidal units with precise positioning spaced by vertical microtubes, shrank and changed into a highly disordered structure (Figure 7b,c upper panel versus Figure 7b,c lower panels). The scaffold structural disorder increased with increasing MNPs content (Figure 7b lower panel versus Figure 7b lower panel). The scaffolds without MNPs showed a different behavior: the seeded cells “opened up” the initial “tent-like” architecture of the scaffolds (Figure 7a upper panel versus Figure 7a lower panel).

The qualitative analysis of the SEM micrographs was confirmed quantitatively by MTS viability assay (Figure 7d). Except the scaffolds without MNPs, where the viability was low for both SMF-stimulated and unstimulated samples, for the scaffolds containing MNPs the relative cells viability was above 75% and increased with increasing MNPs concentration, followed by an additional increase up to 98% following SMF exposure.

For easier visualization of the cell adhesion and morphology on the scaffolds, the insets from Figure 7 were magnified and presented separately in Figure S3 from the Supplementary information file, where, for better viewing, the cells are indicated by red arrows.

#### 3.2.2. Extracellular Matrix Mineralization by Alizarin Red Staining (ARS)

**Figure 8:**
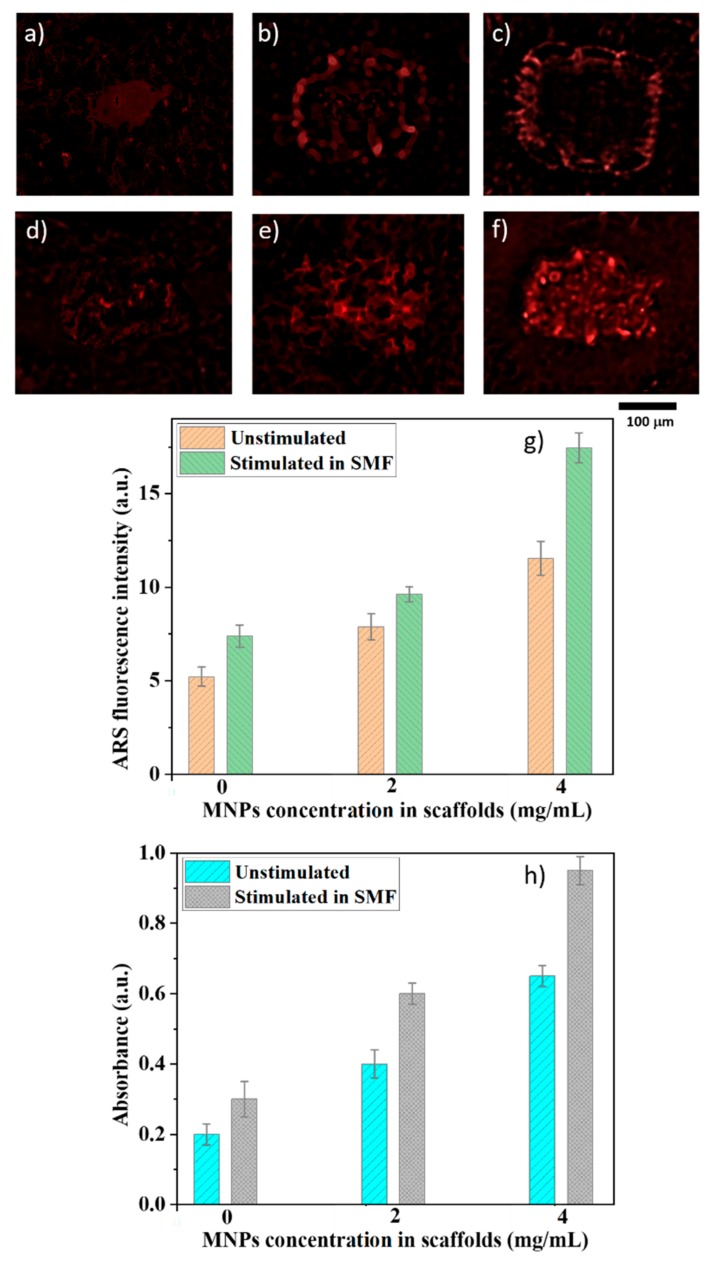
Alizarin Red Staining (ARS) staining of the cell-seeded scaffolds with MNPs concentration of: (**a**,**d**) 0 mg/mL; (**b**,**e**) 2mg/mL; (**c**,**f**) 4 mg/mL, after 20 days of incubation. Upper panel), unstimulated scaffolds. Lower panel, scaffolds exposed to SMF; (**g**) ARS fluorescence intensity as determined by ImageJ; (**h**) absorbance measurements for ARS marking of the mineral deposits in cells growing on scaffolds with different MNPs concentrations (except for the scaffolds without MNPs, the results were statistically significant (*p* < 0.05)).

ARS was monitored by fluorescence microscopy for detecting the presence of calcium in the cellular deposits, which is generally used as indicative of early matrix mineralization [37]. The ARS fluorescence intensity increased with increasing MNPs concentration in the scaffolds, indicating more mineralized deposits (Figure 8a–c). The presence of SFM further increased the fluorescence signal (Figure 8d–f), proving the positive role of magnetic stimulation for early extracellular matrix mineralization. The ARS fluorescence intensity was measured using ImageJ software and supports the above findings (Figure 8g). Except for the scaffolds without MNPs, the results were statistically significant, indicating a significant increase of the mineral deposits with increasing MNPs concentration in the scaffolds (Figure 8h). Further increase of the mineral deposits was observed in the samples exposed to SMF i.e., an increase of the mineral deposits up to 50% was found in the cell-seeded scaffolds with MNPs concentration of 4 mg/mL.

## 4. Discussion

The attempts to control the cellular behavior in magnetic scaffolds face the major challenge of fabricating 3D structures with controlled architectures and homogenous distribution of the MNPs in the whole volume of the scaffold [23]. In the present study, we report the fabrication of 3D magnetic scaffolds with submicronic spatial resolution, high reproducibility and uniform distribution of the MNPs in the whole scaffolds structure, that promote the cell attachment and early mineralization under static magnetic field (SMF) stimulation. The scaffolds were fabricated by laser direct writing via two photons polymerization (LDW via TPP) of Ormocore/MNPs composites. The MNPs with diameters of 4.9 ± 1.5 nm were added to the photopolymer in concentrations of 0, 2 and 4 mg/mL. 

In this paper, we brought several major improvements as compared to the fabrication methods used thus far. This is the first time that LDW via TPP is used for building magnetic scaffolds, which brings significant advantages over the methods previously employed. One is that LDW via TPP technique has undoubtable superiority as compared to other techniques used thus far, in terms of high spatial resolution of about 90 nm [43] and full reproducibility of the structures, which are both essential for systematic in vitro studies. Moreover, the MNPs were directly incorporated into the scaffolds during the photopolymerization process, without any additional processing steps. Most importantly, the homogenous distribution and the superparamagnetic behavior of the MNPs from the unpolymerized composite were preserved after the photopolymerization process, with MNPs uniformly dispersed within the entire structure of the scaffolds. The MNPs also improved the mechanical resilience of the scaffolds by significant reduction of the scaffolds’ shrinkage. Of course, one must also keep in mind the limitations of the technique, such as long production time for large volume fabrication for scaffolds to be used clinically. Additionally, the expected mechanical stability of the scaffolds at large volume should be investigated. Moreover, to assess the origin of the SMF effects on the cellular behavior, controlled and quantitative biological investigations are required.

The lack of cellular toxicity of the MNPs in the presence of a magnetic field has been already proven [6]; therefore, we could consider that there are practically no limitations concerning the number of MNPs from the biological point of view. In our experimental conditions, the MNPs concentration and thus the number of MNPs per ellipsoidal unit of the scaffold was selected based on a tradeoff: on one side, we had to obtain a magnetic response form the scaffolds during exposure to SMF and thus a high enough number of MNPs was required; on the other side, the number of MNPs had to be low enough for allowing the photopolymerization process, since, as we state in the Results section, concentrations of MNPs higher that 4 mg/mL impeded the photopolymerization.

In the absence of MNPs, the scaffolds collapsed and shrunk in the shape of a tent (Figure 2a). As the concentration of MNPs in the composite increased, the shrinkage of the scaffolds became less significant (Figure 2b,c). The basis of the scaffolds was not collapsing, as the substrate adherence was sufficient to hold the scaffolds in place. 

The shrinkage is a serious problem when fabricating micro/nanofeatured structures over a large area. This is caused mainly by the material densification as compared to the material before polymerization and results in volume reduction [44]. The shrinkage depends strongly on the type of architecture, since the geometrical deformations appear when the structure has not sufficient rigidity to withstand the developing and drying process. Another important factor is the hardness of the bulk material to be polymerized. Over the last years, there were several attempts to synthetize photopolymerizable materials with ultra-low shrinkage and negligible geometrical distortions during the development, by introducing in the photopolymer non-linear chromophores, quantum dots or organic dyes, for photonics and metamaterial production [44,45]. Within this context, our experimental results indicate that the MNPs added to the Ormocore reinforced the scaffolds’ structure.

In general, the influence of the matrix stiffness on the cell behavior cannot be excluded. The facts that the dimensions of the scaffolds are very small i.e., of the order of hundreds if m^3^ and that their architecture is very complex make their mechanical characterization very difficult by standard methods. Instead, what we can certainly state in the particular case of our experimental conditions is that the photopolymer used for building the scaffolds (Ormocore) has high mechanical and chemical stability, as reported by the producer (Micro resist technology GmbH, Berlin, Germany). Additionally, given that the MNPs concentration in the scaffolds was very low, their influence on scaffold stiffness when the scaffolds were exposed to SMF is less to be expected. Moreover, to demonstrate that the SMF exposure does not change the stiffness of the scaffolds, we recorded SEM images of scaffolds immediately after the fabrication process and after 20 days of exposure to SMF of 1.3 T. (Figure S2 in the Supplementary information file shows an example for a scaffold with MNPs concentration of 4 mg/mL, but the same observation stands for the 0 and 2 mg/mL concentrations that were used in our study).

Further investigations by enhanced dark field microscopy, magnetic force microscopy and Energy-Dispersive X-ray Spectroscopy showed that the MNPs were uniformly dispersed in the entire structure of the scaffolds (Figure 3 and Figure 4b), preserved the stoichiometry of the composite (Table 1 and Table 2) and retained their superparamagnetic behavior (Figure 6).

We also investigated the functionality of the scaffolds by assessing the effect of an externally applied static magnetic field (SMF) of 1.3 T on the cells behavior, in terms of cells attachment and extracellular matrix mineralization. 

The magnetic field of 1.3 T was provided by the magnets used in our experimental conditions (as described in the Experimental section). We considered that this value of the magnetic field strength is appropriate for the experiments based on the fact that previous studies with significant relevance have already proven the ability of SMF of the order of 1.2 T to control the cells behavior [26,27]. In addition, one must underline that the main point of interest in not the strength of the SMF, but rather the magnetic field gradient is the main factor accounting for the cellular behavior in experimental conditions similar as ours. For example, a SMF of approximately 1 T with a large gradient (up to 1 GT/m) generated by micromagnet arrays was capable of assisting the cells migration [27], having a significant impact on the biological functionality of the cells [26]. Similarly with previous studies on magnetic scaffolds exposed to SMFs, in the superparamagnetic scaffolds reported in our study the MNPs acted as field concentrators of the SMF and produced high gradients magnetic fields within the cells bodies [26,27] that further promoted the cells differentiation process [24,28].

The study was carried out comparatively with scaffolds unexposed to SMF. In order to discriminate the influence of the scaffolds architecture form that of the magnetic field, scaffolds without MNPs were also investigated. It is worth mentioning that, although the concentrations up to 4 mg/mL used in this study were higher than those tested in previous works [5], the scaffolds provided a biocompatible 3D environment for the seeded cells as shown by SEM investigations (Figure 7). 

On all the scaffolds from our study, regardless of the presence or the absence of SMF, the cells were stretched and had a mature osteoblast phenotype similar with the one from the bone surface [46]. The number of attached cells increased with increasing MNPs concentration (Figure 7). Given the superparamagnetic behavior of these MNPs that excludes the presence of magnetic forces in the absence of an external magnetic field, this trend can be attributed to the nanostructuring of scaffolds surfaces (insets from Figure 2) that increased the surface area and provided mode contact points for focal adhesions. 

For the scaffolds without MNPs, the effect of SMF on the cell attachment was not significant. In contrast, on the scaffolds with 2 and 4 mg/mL MNPs concentrations, the applied SMF increased significantly the number of the attached cells (Figure 7b,c upper panels versus Figure 7b,c lower panels). For the scaffolds with 4 mg/mL MNPs concentration, the cells were even able to penetrate down to the inner parts of the structure where they formed an interlaced fibrous network (Figure 7c lower panel). 

An interesting finding was that all cell-seeded scaffolds were highly deformed when exposed to SMF. The scaffolds’ architecture changed from regular ellipsoidal units with precise positioning, characteristic for the unstimulated samples, to a highly disordered architecture (Figure 7a–c upper panels versus Figure 7a–c lower panels). The scaffolds’ deformation increased with increasing MNPs concentration. The reason for the “opening-up” of the scaffolds without MNPs under SMF exposure (Figure 7a lower panel) is yet unknown. A possible explanation could be the absence of high gradient fields in these samples, which eliminates the role of magnetic forces in modulating the cells behavior [27].

The existing studies and the comparative analysis of the relationship between magnetic scaffolds and cell behavior remain unresolved because of the diversity in scaffolds architectures, theoretical models and investigation methods. In general, the influence of cells on scaffolds are described in terms of the contractile forces they generate, which further induce deformations of the scaffolds’ structure [47,48,49]. Several studies succeeded to guide the establishment of cell networks via cellular response to high gradients magnetic fields [26,27]. Positive influence of external static magnetic field on magnetic nanoparticle-incorporated scaffolds on osteoblast differentiation and bone formation has been reported [24]. The MNPs acted as concentrators of the externally applied magnetic field and generated high gradient magnetic fields over the cells bodies, thus modulating their behavior [26,27].

In our experimental conditions, the MNPs added to the scaffolds enhanced the magnetic response, as investigated by VSM magnetometry. The scaffolds with 2 mg/mL and 4 mg/mL MNPs concentrations have both shown a detectable magnetization signal (10^−4^ emu) (Figure 6). The calculated specific magnetization yielded good results that are in agreement with the nanoparticles counting per scaffold, specifically 14 and 17 emu/g for scaffolds with 2 and 4 mg/mL MNPs concentrations, respectively. One must keep in mind that the magnetic properties of the composite scaffolds are determined by the size and magnetization of the MNPs, by their homogenization in the photopolymer and by the porous structure of the scaffolds [24].

**Figure 9:**
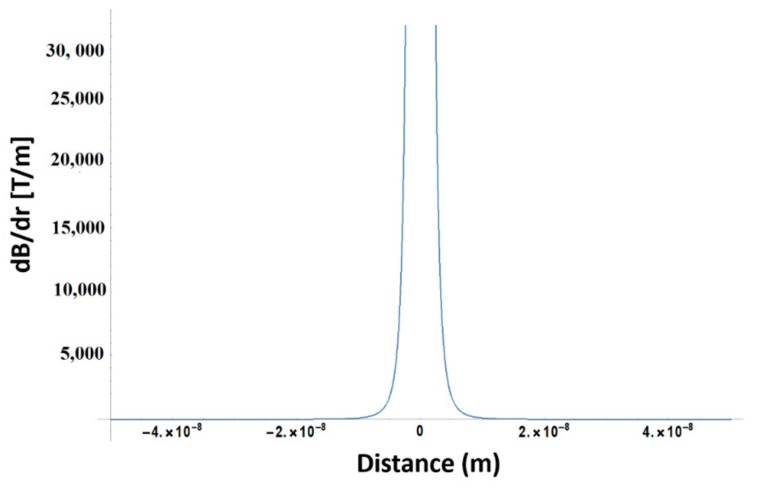
Magnetic field gradient between two adjacent MNPs.

In order to compute the field gradient generated by the MNPs along a distance (r), we employed the following formula [26]:

(1) $$\frac{dB}{dr}=\frac{2\mu_{0}M_{S}R^{2}}{r^{4}}$$

where *M_S_* is the saturation magnetization, *R* is the MNP radius and *μ*_0_ = 4 × 10^−7^ H/m is the vacuum permeability. For our case, *Ms* ≈ 30 emu/g, *R_MNP_* = 2.45 nm. For 2 mg/mL and 4 mg/mL concentration, given that ρ_γ-Fe2O3_ = 4.86 g/cm^3^, the average distance between two nanoparticles is of 50.8 and 40.3 nm, respectively. According with Equation (1), the field gradient between two adjacent MNPs in the first approximation limit is of the order of 3 × 10^4^ T/m and is presented in Figure 9.

Under high magnetic gradients, the cells are subjected to magnetic compressive or tensile stresses that cause membrane deformation, reorganization of the cytoskeleton and increase the tension of the actin filaments [26]. It is known that in moderate magnetic fields with gradient larger than 10^4^ T/m the magnetic force magnitudes are comparable with those of gravity and are sufficient to affect the cell machinery [26,27]. Within this framework, the field gradients reached in our experimental conditions explain the preferential cell attachment and the dramatic changes of the scaffolds architecture for the cell-seeded scaffolds exposed to SMF. The fact that the cells attachment and the scaffolds’ deformation increased with increasing MNPs concentration were likely caused by the higher magnetic field gradients exerting stronger magnetic stresses on the cells. The disordered scaffold structure induced by the SMF exposure and the anisotropy of the structural changes observed in Figure 2a–c lower panels are likely determined by the distribution of the magnetic gradient across the cell volume.

To validate the proposed concept, we monitored the extracellular matrix mineralization for cells seeded on the scaffolds. For this, we employed alizarin that emits a red signal under fluorescent green light and has been widely used for detailed identification of early mineralization events, with a good signal/noise ratio [37]. Alizarin detected under fluorescence at the absorbance at 405 nm increased with increasing MNPs concentration (Figure 8g,h), which confirms the presence of more mineralized deposits in these samples. The fluorescence intensity and the 405 nm absorbance were further increased by SMF exposure, indicating that the applied magnetic field fastened the extracellular matrix mineralization.

Our experimental results provide evidence that the static magnetic field and the magnetic scaffolds acted in synergy and generated favorable conditions for bone cells attachment and early mineralization. These findings complete the diverse scenarios reported by previous studies. SMF stimulation with 0.4 T of osteoblasts seeded on magnetic scaffolds resulted in an increase in the Alkaline Phosphatase activity and induced changes in cell morphology [49]. Human osteosarcoma cells seeded on poly(l-lactic acid) scaffolds exposed to SMF had a more differentiated phenotype depending on cell type and field strength [8]. Polycaprolactone/magnetic nanoparticles scaffolds used in combination with SMF stimulated the osteoblasts to reach a mature stage earlier and to deposit mineral phase more rapidly [24]. 

Given that the architectures of the scaffolds, the MNPs concentrations and their distributions inside the scaffolds differ between those studies and were much less controllable than in our experimental conditions, a straight comparison between previously published results and those reported by the present study are not straightforward. Nevertheless, our results provide evidence that the stronger deformation of the cell-seeded scaffolds and the faster cell mineralization with increasing MNPs concentration in the scaffolds exposed to SMF are due to local effects of magnetic forces.

Preliminary in vivo studies are currently carried out (Figure S4 from the Supplementary information). Wistar rats with scaffolds having 4 mg/mL MNPs concentration and implanted at femoral level were maintained in the static magnetic field by placing two powerful magnets under the cages in which they were accommodated. Images of computed tomography (CT) recorded at different time points after the implantation procedure did not highlight inflammatory processes. At 15 days post-implantation, for both SMF and non-stimulated groups, the CT evaluation showed that the scaffolds were in the right position, without signs of hematoma, edema or infection. Tissue necrosis has not been detected. The bone tissue was visible around the scaffolds, providing evidence that the scaffolds had a strong osteointegration. Importantly, the groups stimulated in SFM have shown a faster bone regeneration than the unstimulated specimens. These preliminary results provide evidence about the histocompatibility of these new magnetic scaffolds that have been implanted for the first time in vivo and provide great potential for the development of a long-term in vivo study. The results and the conclusions of the in vivo study will be the subject of a future report.

## 5. Conclusions

We designed and built homogeneous 3D superparamagnetic scaffolds and we proved their potential for biological applications. The scaffolds were fabricated by laser direct writing via two photons polymerization (LDW via TPP) of Ormocore/magnetic nanoparticles (MNPs) composites. The proposed concept provided unique advantages that were not achievable with other methods and materials. The LDW via TPP technique allowed us to fabricate scaffolds with complex and controlled 3D architectures, with MNPs directly incorporated into the scaffolds during the photopolymerization process. The LDW via TPP process was carried out on a homogenous dispersion of MNPs of pre-established concentrations in Ormocore of 0, 2 and 4 mg/mL. The homogenous distribution and the superparamagnetic behavior of the MNPs from the unpolymerized composite were preserved after the photopolymerization process. A uniform dispersion of the MNPs within the entire structure of the scaffolds was obtained. The MNPs also improved the mechanical resilience of the scaffolds by significant reduction of the scaffolds’ shrinkage. An enhanced magnetic response (10^−4^ emu) has been obtained for all scaffolds containing MNPs, as seen in VSM magnetization measurements. Moreover, the specific magnetizations were found to be in agreement with the nanoparticles counting per scaffold. The intrinsically magnetic cues represented by the MNPs incorporated in the scaffolds acted in synergy with the externally magnetic cues represented by a SMF of 1.3 T and promoted the attachment and the early stage mineralization of nanoparticle-free osteoblast-like cells. The stronger scaffolds’ deformation and the faster extracellular matrix mineralization occurred in the scaffolds having the highest MNPs concentration. The results were explained in the frame of high gradient magnetic fields of the order of 3 × 10^−4^ T/m locally generated by the MNPs over the cells bodies. The proposed method is suitable for other applications that require remote manipulation of magnetic field with submicronic resolution, with great potential for magnetically-driven tissue regeneration.

## Figures and Tables

**Table 1:** Scaffolds shrinkage, number of MNPs from an ellipsoidal unit of a scaffold as determined from enhanced dark field microscopy images using the dedicated plug-ins for nanoparticles counting and scaffolds porosity determined as in [36].

MNPs Concentration in the Unpolimerized Composite (mg/mL)	Scaffolds Shrinkage (%)	Number of MNPs from an Ellipsoidal Unit of a Scaffold	Scaffolds Porosity (%)
0	80.2 ± 5.3	0	46.2 ± 4
2	35.6 ± 4.2	178 ± 5	87.6 ± 2
4	20.7 ± 4.7	332 ± 8	94.6 ± 1

**Table 2:** Elemental composition of the polymerized Ormocore/MNPs composites as determined by EDS.

MNPs Concentration in Unpolymerized Composite (mg/mL)	Element	Atomic %	Error %
**0**	C K	73.6	4.7
	O K	25.4	7.4
	Fe L	0.01	-
**2**	C K	75.1	4.7
	O K	23.7	7.4
	Fe L	1.2	6.6
**4**	C K	69.0	5.0
	O K	28.3	7.1
	Fe L	2.7	6.2

## Supplementary Materials

The following are available online at https://www.mdpi.com/1996-1944/12/17/2834/s1, Figure S1: Optical images of the laser irradiated material at the laser focus, at different time points during the laser direct writing process: (a) at the beginning of the process, the laser spot is visible on the sample; (b) bubble formation at the irradiation spot, after few seconds of interaction of the focused laser beam with the material; (c) local micro-explosion at the laser-material interaction spot. Figure S2: SEM micrographs of a scaffold with MNPs concentration of 4 mg/mL: (a) immediately after the fabrication process and (b) after 20 days of exposure to SMF of 1.3 T. Figure S3: SEM micrographs illustrating cells attached on scaffolds with MNPs concentrations of: (a) 0 mg/mL; (b) 2 mg/mL; (c) 4 mg/mL, after 3 days of cultivation, in the absence (upper panel) and in the presence (lower panel) of SMF. The cells are indicated by red arrows. Figure S4: Preliminary results in vivo: CT scans of Wistar rats with scaffolds implanted at femoral level, at different time points after the implantation procedure.
