# Supplementary material for: 3D Superparamagnetic Scaffolds for Bone Mineralization under Static Magnetic Field Stimulation
Source: Materials (Basel). 2019 Sep 3;12(17):2834. doi: 10.3390/ma12172834 (PMC6747966; doi:10.3390/ma12172834)
Supplement: Supplementary file 1 [file materials-12-02834-s001.pdf]

materials

MDPI

Supplementary Information

3D Superparamagnetic Scaffolds for Bone Mineralization under Static Magnetic Field Stimulation

Irina Alexandra Paun [1,2,*], Bogdan Stefanita Calin [1,2], Cosmin Catalin Mustaciosu [3], Mona Mihailescu [2], Antoniu Moldovan [4], Ovidiu Crisan [4], Aurel Leca [4] and Catalin Romeo Luculescu [1]

[1] Center for Advanced Laser Technologies (CETAL), National Institute for Laser, Plasma and Radiation Physics, RO-077125 Magurele-Ilfov, Romania
[2] Physics Department, Faculty of Applied Sciences, University Politehnica of Bucharest, RO-060042 Bucharest, Romania
[3] Horia Hulubei National Institute for Physics and Nuclear Engineering IFIN-HH, RO-077125 Magurele-Ilfov, Romania
[4] National Institute for Laser, Plasma and Radiation Physics, RO-077125 Magurele-Ilfov, Romania
[5] National Institute of Materials Physics, RO-077125 Magurele-Ilfov, Romania
* Correspondence: irina.paun@inflpr.ro

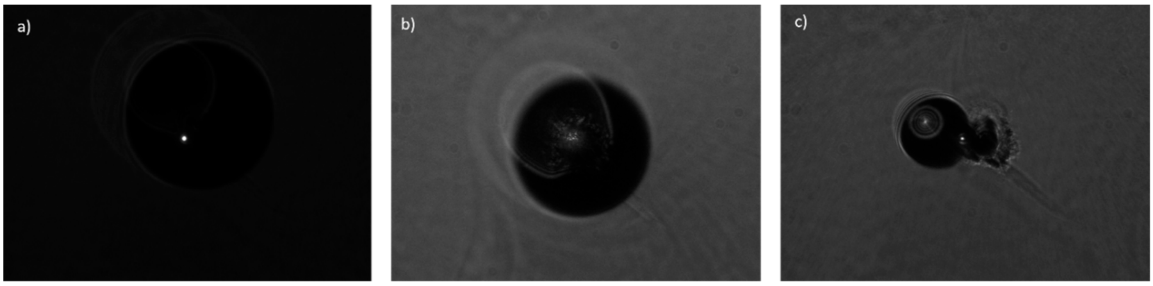

Figure S1. Optical images of the laser irradiated material at the laser focus, at different time points during the laser direct writing process: (**a**) at the beginning of the process, the laser spot is visible on the sample; (**b**) bubble formation at the irradiation spot, after few seconds of interaction of the focused laser beam with the material; (**c**) local micro-explosion at the laser-material interaction spot.

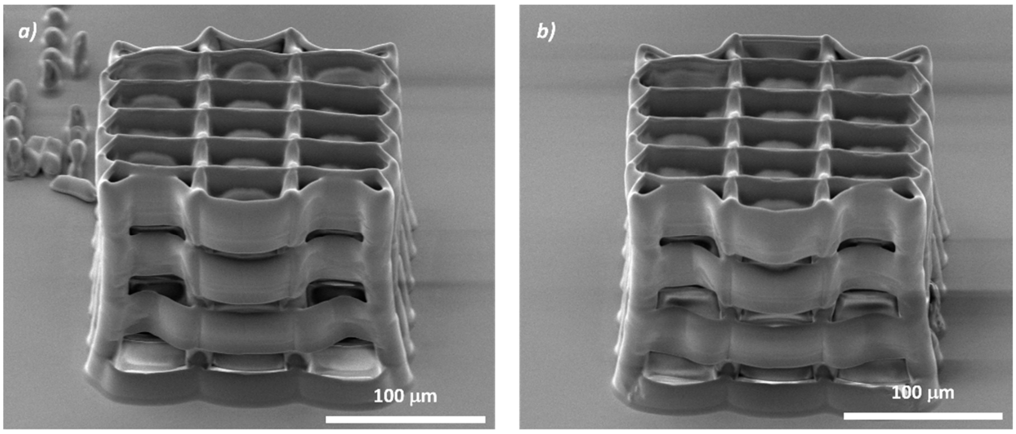

Figure S2. SEM micrographs of a scaffold with MNPs concentration of 4 mg/mL: (**a**) immediately after the fabrication process and (**b**) after 20 days of exposure to SMF of 1.3 T.

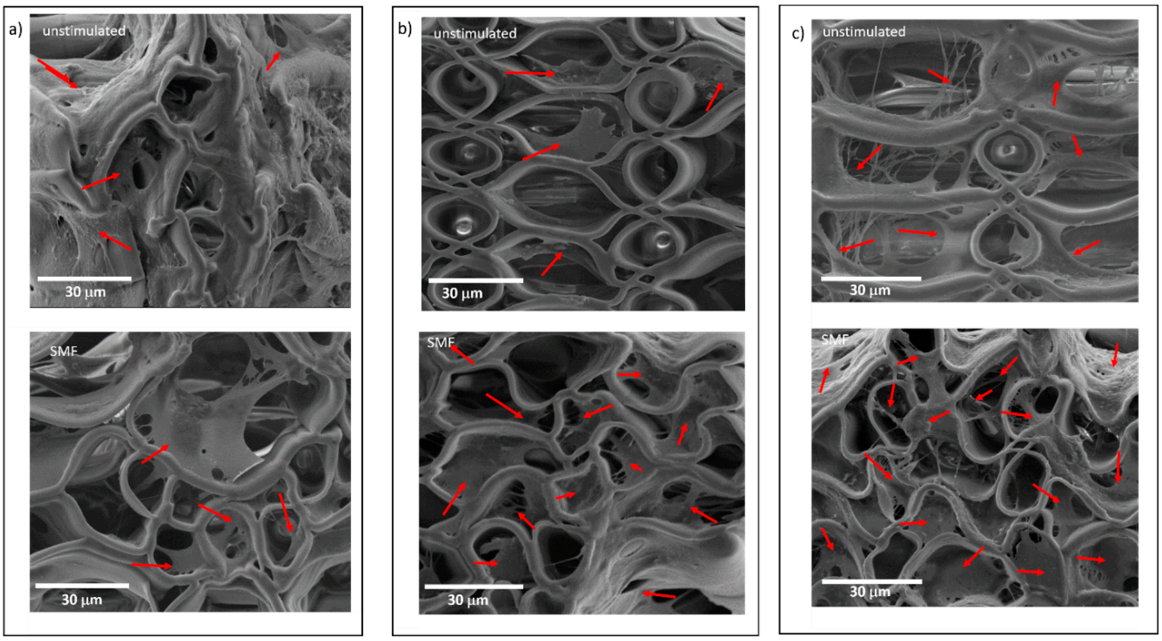

Figure S3. SEM micrographs illustrating cells attached on scaffolds with MNPs concentrations of: (**a**) 0 mg/mL; (**b**) 2 mg/mL; (**c**) 4 mg/mL, after 3 days of cultivation, in the absence (upper panel) and in the presence (lower panel) of SMF. The cells are indicated by red arrows.

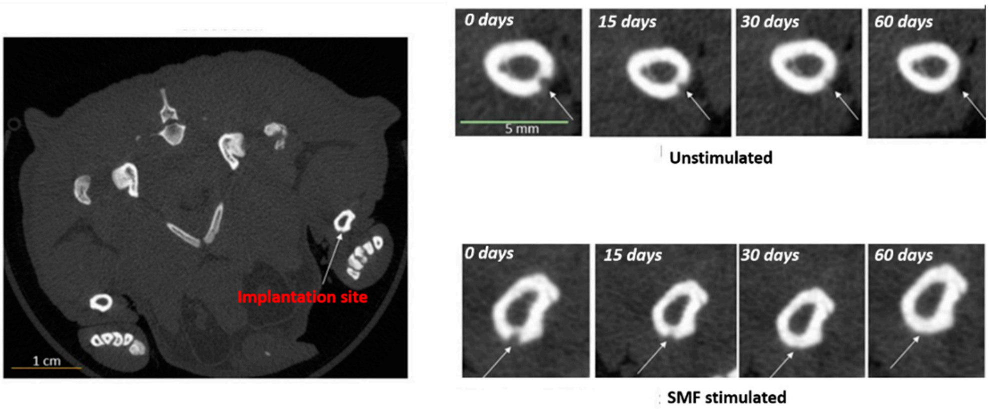

Figure S4. Preliminary results in vivo: CT scans of Wistar rats with scaffolds implanted at femoral level, at different time points after the implantation procedure.
